# Benzimidazole Derivatives Suppress Fusarium Wilt Disease via Interaction with *ERG6* of *Fusarium equiseti* and Activation of the Antioxidant Defense System of Pepper Plants

**DOI:** 10.3390/jof9020244

**Published:** 2023-02-12

**Authors:** Asmaa El-Nagar, Abdelnaser A. Elzaawely, Hassan M. El-Zahaby, Tran Dang Xuan, Tran Dang Khanh, Mohamed Gaber, Nadia El-Wakeil, Yusif El-Sayed, Yasser Nehela

**Affiliations:** 1 Department of Agricultural Botany, Faculty of Agriculture, Tanta University, Tanta 31527, Egypt; 2 Transdisciplinary Science and Engineering Program, Graduate School of Advanced Science and Engineering, Hiroshima University, Hiroshima 739-8529, Japan; 3 Center for the Planetary Health and Innovation Science (PHIS), The IDEC Institute, Hiroshima University, Hiroshima 739-8529, Japan; 4 Agricultural Genetic Institute, Pham Van Dong Street, Hanoi 122000, Vietnam; 5 Center for Agricultural Innovation, Vietnam National University of Agriculture, Hanoi 131000, Vietnam; 6 Chemistry Department, Faculty of Science, Tanta University, Tanta 31527, Egypt

**Keywords:** *Fusarium equiseti*, wilt disease, metal complex, benzimidazole, *EGR6*, antioxidant, in-silico analysis, molecular docking, bioinformatics

## Abstract

Sweet pepper (*Capsicum annuum* L.), also known as bell pepper, is one of the most widely grown vegetable crops worldwide. It is attacked by numerous phytopathogenic fungi, such as *Fusarium equiseti*, the causal agent of Fusarium wilt disease. In the current study, we proposed two benzimidazole derivatives, including 2-(2-hydroxyphenyl)-1-H benzimidazole (HPBI) and its aluminum complex (Al−HPBI complex), as potential control alternatives to *F. equiseti*. Our findings showed that both compounds demonstrated dose-dependent antifungal activity against *F. equiseti* in vitro and significantly suppressed disease development in pepper plants under greenhouse conditions. According to in silico analysis, the *F. equiseti* genome possesses a predicted Sterol 24-C-methyltransferase (*FeEGR6*) protein that shares a high degree of homology with *EGR6* from *F. oxysporum* (*FoEGR6*). It is worth mentioning that molecular docking analysis confirmed that both compounds can interact with *FeEGR6* from *F. equiseti* as well as *FoEGR6* from *F. oxysporum.* Moreover, root application of HPBI and its aluminum complex significantly enhanced the enzymatic activities of guaiacol-dependent peroxidases (POX), polyphenol oxidase (PPO), and upregulated four antioxidant-related enzymes, including superoxide dismutase [Cu-Zn] (*CaSOD-Cu*), L-ascorbate peroxidase 1, cytosolic (*CaAPX*), glutathione reductase, chloroplastic (*CaGR*), and monodehydroascorbate reductase (*CaMDHAR*). Additionally, both benzimidazole derivatives induced the accumulation of total soluble phenolics and total soluble flavonoids. Collectively, these findings suggest that the application of HPBI and Al−HPBI complex induce both enzymatic and nonenzymatic antioxidant defense machinery.

## 1. Introduction

Sweet pepper (*Capsicum annuum* L.) is a solanaceous annual plant grown worldwide. It is one of the most significant vegetable crops farmed in Egypt for both domestic and international trade. Moreover, the fruit pepper has a remarkable amount of vitamin C and other minerals [1]. Consequently, enhancing pepper plants’ maximum sustainable yield (MSY) is a priority of modern cropping systems in many countries [2]. The global production of sweet pepper in 2020 reached nearly 36 million tons. China was the largest producer of pepper with 16.7 million tons, followed by Mexico, Indonesia, Turkey, and Spain, respectively. After the aforementioned top-five pepper producers, Egypt was the sixth-largest producer of sweet pepper in the same year, producing approximately 1.1 million tons from 58,402 hectares [3].

Pepper plants are attacked by several fungi, bacteria, nematodes, viruses, and herbivorous insects at any stage of their life, causing serious losses [4]. Fungal phytopathogens are the main threat to pepper plants worldwide. However, root rots and wilt are the most destructive plant diseases in the majority of pepper-producing areas worldwide [4], particularly in Egypt [5,6]. Wilt disease is caused by different species of *Fusarium*, such as *Fusarium equiseti*, *F. oxysporum*, and *F. oxysporum* f.s. *capsici* [5,7,8]. *Fusarium* species are soil-borne thermophilic phytopathogens that cause deterioration of the vascular system and inhibit the transportation of water and nutrients, thus disturbing the physiological processes responsible for achieving the best production and quality [9]. Characteristic symptoms of Fusarium wilt disease include yellowing and wilting of plant leaves as if they were suffering from water stress, followed by the death of young branches and their transformation to brown, and then the death of the entire plant [10]. 

It is essential to note that pepper Fusarium wilt disease prevention begins before the seeds are planted. This protects the plants from disease and reduces crop losses [4]. There were numerous methods for controlling Fusarium wilt, including crop rotation, growing resistant cultivars, solarizing the soil, and biological control [11]. Chemicals such as fungicides and other agrochemicals are the most prevalent means of combating Fusarium wilt disease. Chemical fungicides can eliminate plant diseases, but excessive use harms the environment and promotes the development of fungicide-resistant strains, both of which are extremely hazardous for human and animal health and non-target microorganisms [12]. Therefore, there is a growing demand for alternatives to these chemical fungicides that are non-toxic, cost-effective, and environmentally friendly.

Benzimidazoles and their derivatives are among the most important heterocyclic chemicals, playing an essential role in the design and synthesis of bioactive compounds [13]. Recent interest in benzimidazole compounds has increased due to their wide range of biological effects in agricultural and medical chemistry [14,15]. The effectiveness of benzimidazole compounds might be due to their inhibitory activity and their favorable selectivity ratio [16,17]. Various substituted benzimidazole derivatives were proposed to be associated with a wide range of biological activities, including anticancer, antiviral, antibacterial, antifungal, anti-inflammatory, and antioxidant activities [17,18]. Moreover, transition metal complexes of benzimidazole ligand showed strong in vitro antifungal activity against three soil-borne fungi including *F. solani*, *Rhizoctonia solani,* and *Sclerotium rolfesii* [19]. However, the physiological, biochemical, and molecular mechanisms underlying how transition metal chelates boost the biological potentials of their parent ligands are poorly understood. 

Minerals are unquestionably essential for plants to develop effectively [20]. The use of benzimidazole as a ligand for transition metal ions with a variety of biological molecules, including ion heme systems, vitamin B_12,_ and its derivatives, and various metal lipoproteins is very common [21]. Furthermore, the complexes of metal salts with benzimidazole derivatives have been extensively investigated as a model structure of numerous key biological molecules [20]. It has been reported previously that, occasionally, biologically relevant ligands are more active in metal complexes than in the free form [22]. Recently, we demonstrated that the metal complexation of bis-chalcone derivatives enhances their antifungal activity against soil-borne phytopathogens [5].

Additionally, benzimidazoles and their derivatives showed potential antifungal and antibacterial properties against phytopathogenic microorganisms. For instance, a series of benzimidazole derivatives were synthesized and tested against phytopathogenic fungi such as *Botrytis cinerea*, *Colletotrichum gloeosporioides*, *F. culmorum*, and *Sclerotinia sclerotiorum* [23,24]. Furthermore, several metal ions such as, cobalt, nickel, copper, zinc, and cadmium were added to the ligand 1H-anthra [1,2-d]imidazol-6,11-dione-2-[2-hydroxyphenyl] to form several complexes that showed antimicrobial activities against a set of Gram-positive and Gram-negative bacteria, as well as fungi [25]. For instance, fourteen novel cobalt (II) or zinc (II) complexes of benzimidazoles showed antibacterial activity against *Escherichia coli* ATCC25922, *Staphylococcus aureus* ATCC 29213, and *Pseudomonas aeruginosa* ATCC 27853 [22].

In the current study, in vitro antifungal activities of 2-(2-hydroxyphenyl)-1H-benzimidazole (HPBI) and its aluminum complex (Al−HPBI complex) against *F. equiseti*, the causal agent of pepper Fusarium wilt disease, were investigated. Furthermore, we used integrated biochemical and transcriptomic approaches to (i) investigate the potential roles of HPBI and Al−HPBI complex in activating pepper response to fungal infection, and (ii) clarify the regulatory role of HPBI and Al−HPBI complex in mitigating the infection-associated antioxidant enzyme activities. In addition, molecular docking analysis was performed to explain the antifungal activities of HPBI or its aluminum complex (Al−HPBI complex) against *F. equiseti* and to clarify their potential molecular mechanisms via binding with the functional proteins in the causal agent of Fusarium wilt disease.

## 2. Materials and Methods

### 2.1. Tested Compounds

**Figure 1:**
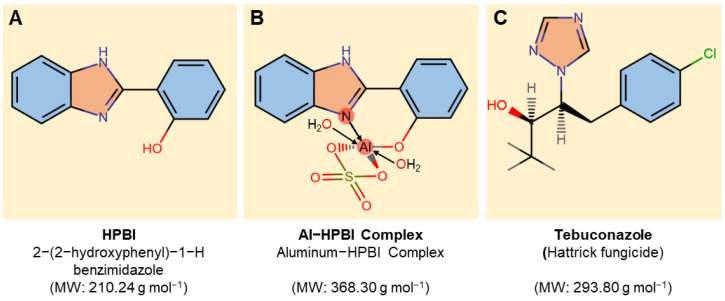
Chemical structure of tested compounds. (**A**) 2-(2-Hydroxyphenyl)-1H-benzimidazole (HPBI), (**B**) aluminum-HPBI complex (Al-HPBI complex), and (**C**) Tebuconazole (Hattrick fungicide). Molecular weight/molar mass (g·mol^−1^) is presented under each compound.

2-(2-Hydroxyphenyl)-1H-benzimidazole (HPBI; Figure 1A) was purchased from Sigma-Aldrich (Darmstadt, Germany), while Al−HPBI complex (Figure 1B) was synthesized and characterized in our previous study [26]. Both compounds (HPBI and Al−HPBI complex) were dissolved first in 5 mL of 100% dimethylformamide (DMF), and then the volume was adjusted to 100 mL using sterilized distilled water to make a 5 mM stock solution. This stock solution was diluted and used in all further experiments. Furthermore, Hattrick fungicide (Tebuconazole 6% FS; (RS)-1-pchlorophenyl 4,4-dimethyl-3-(1H-1,2,4-triazol-1-methyl)pentan-3-ol) was used as a positive control throughout this study at the recommended dose (1 cm^3^. L^−1^ water) (Figure 1C).

### 2.2. Fungal Isolate

*Fusarium equiseti* isolate YN2022 was previously isolated from pepper plants that exhibited typical symptoms of root rot [5], purified using the single-spore technique, identified based on their cultural, morphological, and macroscopic characteristics, and then molecularly identified based on the sequence of their internal transcribed spacer (ITS) region (GenBank accession No. OP339844). The purified cultures of *F. equiseti* were maintained on a sand and barley medium for two weeks at 27 ± 2 °C then used to inoculate 30-cm plastic pots filled with sterilized sandy loam soil (1:1 *w*/*w*), as described in our previous study [5]. The inoculated pots were watered and maintained under greenhouse conditions for two weeks before planting pepper seedlings.

### 2.3. In Vitro Antifungal Activity

The potential antifungal activities of HPBL and Al−HPBI complex were evaluated against *Fusarium equiseti* using the agar diffusion method in vitro [27]. Briefly, an appropriate volume of the stock solution of each compound was added to 20 mL of potato dextrose agar (PDA) medium to create six final concentrations of 0.1, 0.3, 0.5, 0.7, 1, and 2 mM. Hattrick (Tebuconazole 6% FS) was used as a positive control at the standard dose (1 cm^3^/L), and sterilized DMF was used as a negative control at a final concentration of 0.2% in the PDA medium. After media solidification, a 5-mm mycelial plug of newly cultured *F. equiseti* culture was placed on the surface of prepared Petri dishes and incubated at 27 ± 2 °C for seven days until the mycelial growth completely cover the control plate. The mycelial growth inhibition (%) [27] was calculated using Equation (1):

(1) $$\mathrm{Inhibition}\;(\%)=\frac{C-T}{C}\times 100$$

where “*C*” and “*T*” denote the mycelial growth in the negative control plate and treated plate, respectively. The experiment was repeated three times.

### 2.4. Greenhouse Experiment, Disease Assessment, and Growth Parameters

The impact of tested compounds (HPBL and Al−HPBI complex) on pepper plants infected with *F. equiseti* was tested under greenhouse conditions. Briefly, 30-day-old pepper seedlings were dipped for two hours in one of the tested compound solutions before being planted in *F. exquisite*-inoculated pots, as described above. The seedlings were dipped in sterilized distilled water (as a negative control; mock) or Hattrick fungicide (Tebuconazole 6% FS; as a positive control). After transplanting the seedlings, pots were watered once a week, and other standard cultural practices and fertilization were followed as recommended. The whole experiment was repeated twice during the spring seasons of 2020 and 2021. The experiment was carried out using a completely randomized design (CRD) with six biological replicates per treatment and five plants for each replicate.

The disease severity was assessed at 20-, 30-, 40-, 50-, and 60-days post-treatment (dpt) according to the scale suggested by Song et al. [28], and the area under the disease–progress curve (AUDPC) was calculated, as described by Jeger et al. [29]. Furthermore, total chlorophyll (SPAD), plant height (cm), the number of leaves per plant, fresh weight (g plant^−1^), and total leaf area (cm^2^) were assessed as growth indicators at 60 dpt.

### 2.5. In Situ Histochemical Localization of Hydrogen Peroxide (H_2_O_2_) and Superoxide Anion (O_2_^•−^)

Pepper leaves were collected at 24-, 48-, 72-, 96-, and 120-hours post-treatment (hpt) with tested compounds, as well as both controls (Mock and fungicide), to assess hydrogen peroxide (H_2_O_2_) and superoxide anion (O_2_^•−^). H_2_O_2_ was histochemically localized, as described by Romero-Puertas et al. and Shi et al. [30,31], using 3,3-diaminobenzidine (DAB; Sigma-Aldrich, Darmstadt, Germany), with slight modifications, as published previously [32,33], until the development of a brown color. Likewise, in situ histochemical localization of O_2_^•−^ was assessed using nitro blue tetrazolium (NBT; Sigma-Aldrich, Darmstadt, Germany) until the development of a purple color. The intensity of the brown (as an indicator of H_2_O_2_) or purple (as an indicator of O_2_^•−^) colors was determined using the Image J image processing tool, Fiji version (http://fiji.sc; access date 20 June 2022), after bleaching the leaves with 0.15% (*w*/*v*) trichloro acetic acid (TCA) in ethanol:chloroform 4:1 (*v*/*v*) [34].

### 2.6. Total Soluble Phenolic and Flavonoid Compounds

The total soluble phenolics were determined in pepper leaves collected at 24, 48, 72, 96, and 120 hpt with tested compounds, as well as both controls (Mock and fungicide), using the Folin-Ciocalteu reagent (FCR), as described by Kähkönen, et al. [35], with slight modification. Briefly, 100 mg of ground fresh pepper leaves was extracted for 24 h using 20 mL of 80% methanol. Then, 200 μL of methanolic extract was mixed with 1 mL of 10% FCR and vortexed for 30 s. Three minutes later, the mixture received 800 μL of sodium carbonates (*w*/*v*) at 7.5%. The mixture was incubated at room temperature for 30 min, and then absorbance was measured at 765 nm. The total soluble phenolic content was presented as the gallic acid equivalents per gram of fresh weight (mg GAE g^−1^ FW). Additionally, total soluble flavonoids were determined using the methods described by Djeridane, et al. [36]. Briefly, 1 mL of methanolic extract was thoroughly mixed with 1 mL of methanolic aluminum chloride (2%) and incubated at room temperature for 15 min, then absorbance was measured at 430 nm. The total soluble flavonoids were expressed as Rutin equivalents per gram fresh weight (mg RE g^−1^ FW).

### 2.7. Enzymatic Activity

The enzymatic activities of guaiacol-dependent peroxidases (POX) and polyphenol oxidase (PPO) were determined in pepper leaves collected at 24, 48, 72, 96, and 120 hpt with both compounds (HPBL and Al−HPBI complex), as well as both controls (Mock and fungicide). Briefly, approximately 500 mg of pepper leaf tissues were homogenized using a pre-cooled mortar and pestle that contained 3 mL of 50 mM Tris buffer (pH 7.8) containing 1 mM EDTA-Na_2_ and 7.5% polyvinylpyrrolidone. The homogenate was then centrifuged at 4 °C for 20 min at 12,000 rpm. For the POX activity, about 100 µL of crude enzyme extract was reacted with 100 µL of guaiacol, 100 µL of 12 mM H_2_O_2_, and 2.2 mL of 100 mM sodium phosphate buffer (pH 6.0). The conjugate’s development was tracked by a rise in the absorbance at 436 nm using an extinction coefficient of 26.6 mM^−1^ cm^−1^ [37]. For PPO activity, about 100 µL of crude enzyme extract was added to 3 mL catechol solution (0.01 M), freshly prepared in 0.1 M phosphate buffer (pH 6.0). The variations in absorbance at 495 nm were recorded every 30 s for 3 min [38].

### 2.8. Gene Expression Analysis

The expression levels of four antioxidant-related genes, including superoxide dismutase [Cu-Zn] (*CaSOD-Cu*; GenBank accession No. NM_001398340.1), L-ascorbate peroxidase 1, cytosolic (*CaAPX*; GenBank accession No. NM_001325037.1), glutathione reductase, chloroplastic (*CaGR*; GenBank accession No. XM_016710630.2), and monodehydroascorbate reductase (*CaMDHAR*; GenBank accession No. XM_016687442.2) were determined from pepper leaves at 72 hpt using real-time RT-PCR. Briefly, genomic RNA was extracted using a Simply P Total RNA Extraction Kit (catalog number BSC52S1), according to the manufacturer’s protocol. The quality and quantity of extracted RNA were measured using the A NanoDrop 2000 spectrophotometer (Thermo Scientific, Waltham, MA, USA). Subsequently, cDNA was synthesized using the TOP script^TM^ cDNA Synthesis Kit according to the manufacturer’s protocol. The reaction, thermo cycles, and sequences of the primers used for the RT-PCR analysis were performed as described in our previous study [5]. Actin was used as a housekeeping gene for the normalization of gene expression. The relative gene expressions were calculated using the 2^−ΔΔCT^ method [39]. Gene expression was analyzed from two technical replicates of six biological replicates per treatment (*n* = 12), as described in our previous studies [40,41,42,43].

### 2.9. In Silico Analysis of EGR6 from F. equiseti

#### 2.9.1. Protein–Protein BLAST (BLASTp) Analysis

Sterol 24-C-methyltransferase (*FoEGR6*; GenBank accession no. XP_031038350.1; 382 aa) from *Fusarium oxysporum* NRRL 32931 was used as a query sequence to identify putative *FeEGR6* candidates from *F. equiseti* using the Basic Local Alignment Search tool (BLAST), particularly the protein–protein BLAST (BLASTp 2.8.0+) algorithm, based on recently available data on GenBank, National Center for Biotechnology information website (NCBI, http://www.ncbi.nlm.nih.gov/gene/, accessed 22 October 2021), using the compositionally adjusted substitution matrices [44]. Subsequently, a shortlist of top matches was generated (Table S1) based on the query cover (%) and identity (%) of more than 90%.

#### 2.9.2. Multiple Sequence Alignment Analysis

Amino acid sequences of putative *FeEGR6* candidates from *F. equiseti* that produced significant homology with *FoEGR6* from *F. oxysporum* NRRL 32931 were simultaneously aligned using the Constraint Based Alignment tool (COBALT) [45]. Furthermore, the top-matched sequence of *FeEGR6* from *F. equiseti* (GenBank accession no. CAG7563035.1; 381 aa) producing significant alignments with *FoEGR6* from *F. oxysporum* NRRL 32931 was used to generate the multiple sequence alignment using ClustalW tool [46,47], and the conserved regions in the alignment output were visualized using BOXSHADE-version 3.21.

#### 2.9.3. Conserved Domains and Theoretical pI/Mw

The functionally important domains and conserved sites of the top-matched sequence of *FeEGR6* from *F. equiseti* and *FoEGR6* from *F. oxysporum* NRRL 32931was interactively identified using InterPro tool (https://www.ebi.ac.uk/interpro/, accessed 24 October 2021) [48]. Additionally, the theoretical isoelectric point (pI) and molecular weight (MW) of putative *FeEGR6* candidates from *F. equiseti* were computed using the Compute pI/Mw tool (http://web.expasy.org/compute_pi, accessed 24 October 2021) [49].

#### 2.9.4. Three-Dimensional (3D) Structure Modeling

The three-dimensional (3D) structure of *FeEGR6* from *F. equiseti* and *FoEGR6* from *F. oxysporum* NRRL 32931 was generated using the SWISS-MODEL server (https://swissmodel.expasy.org/, accessed 24 October 2021) [50], using the crystal structure of 4’-O-methyltransferase (*RebM*; PDB ID 3BUS.1.A) as a template. The predicted 3D structures of *EGR6* (PDB format) were interactively visualized using the UCSF-Chimera package [51].

### 2.10. Molecular Docking Analysis

Molecular docking between the 3D models of putative *FeEGR6* protein from *F. equiseti* or *FoEGR6* protein from *F. oxysporum* NRRL 32931 and both tested compounds (HPBL and Al−HPBI complex), as well as Hattrick fungicide, were investigated using the Molecular Operating Environment (MOE) software. Around the protein, all the water molecules were eliminated, and hydrogen was added. The form of each examined molecule with the minimum binding energy was subsequently optimized using the MMFF94x force field [52,53]. Alpha-site spheres were generated using the site finder of the MOE software. On a personal computer of Intel^(R)^ Core^TM^ i5-5300U CPU @ 2.30 GHz, the GAUSSIAN 09 w software package was utilized for DFT simulations [54]. For the optimization of synthetic compounds, a hybrid B3LYP functionality was used [55]. The geometry of the ligand structure was optimized by using B3LYP/6-31++G(d,p) basis set. The binding free energy (Kcal·mol^−1^), which is a measure of the binding affinity, was calculated from the hydrogen bonds formed between the compounds and proteins. By docking the cocrystalline ligand and its aluminum complex (Al−HPBI complex), we verified our docking methodology and obtained the root mean square deviation (RMSD) values in the range of 1.3161 to 3.1458 Å, and strain energy values in the range 39.06–40.50 Kcal·mol^−1^.

### 2.11. Statistical Analyses

Throughout this study, all experiments were conducted during two successive seasons (2020 and 2021) using a completely randomized design (CRD) with six biological replicates per treatment and five plants for each replicate. All data were analyzed according to the analysis of variance (ANOVA) test, followed by Tukey-Kramer honestly significant difference test (Tukey HSD; *p* ≤ 0.05) as a post hoc analysis for pairwise comparisons [56].

## 3. Results

### 3.1. HPBI and Al−HPBI Complex Suppress the Mycelial Growth of F. equiseti

**Figure 2:**
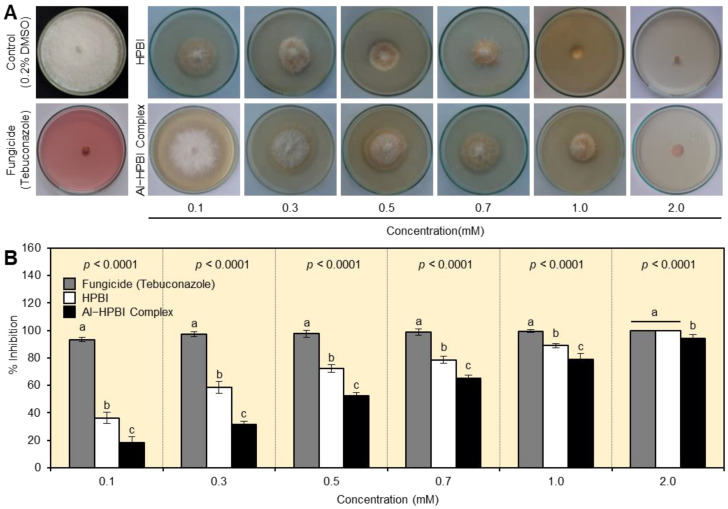
In vitro antifungal activity of HPBI and its aluminum complex against *Fusarium equiseti*. (**A**) Antifungal activity of HPBI and its aluminum complex against *F. equiseti*. (**B**) Inhibition (%) of mycelial growth of *F. equiseti* growing on PDA medium containing HPBI or its aluminum complex. Bars represent the means ± standard deviation (means ± SD) of six biological replicates (*n* = 6). Different letters signify statistically significant differences between treatments (*p* < 0.05). The experiment was repeated twice with similar results.

The antifungal activities of both HPBI and Al−HPBI complex against *F. equiseti* were tested in vitro at six different concentrations (0.1, 0.3, 0.5, 0.7, 1.0, and 2.0 mM). Both compounds showed strong dose-dependent fungistatic activity and significantly inhibited the mycelial radial growth of *F. equiseti* (Figure 2A). It is worth mentioning that the complexation of HPBI with aluminum significantly reduced its antifungal activity at all tested concentrations (Figure 2A,B). At a high concentration (2 mM), HPBI was similar to the positive control (fungicide) and significantly inhibited the mycelial growth of *F. equiseti* by 100%, followed by Al−HPBI complex (94.26%; Figure 2B).

### 3.2. HPBI and Al−HPBI Complex Reduce the Development of Fusarium Wilt Disease

**Figure 3:**
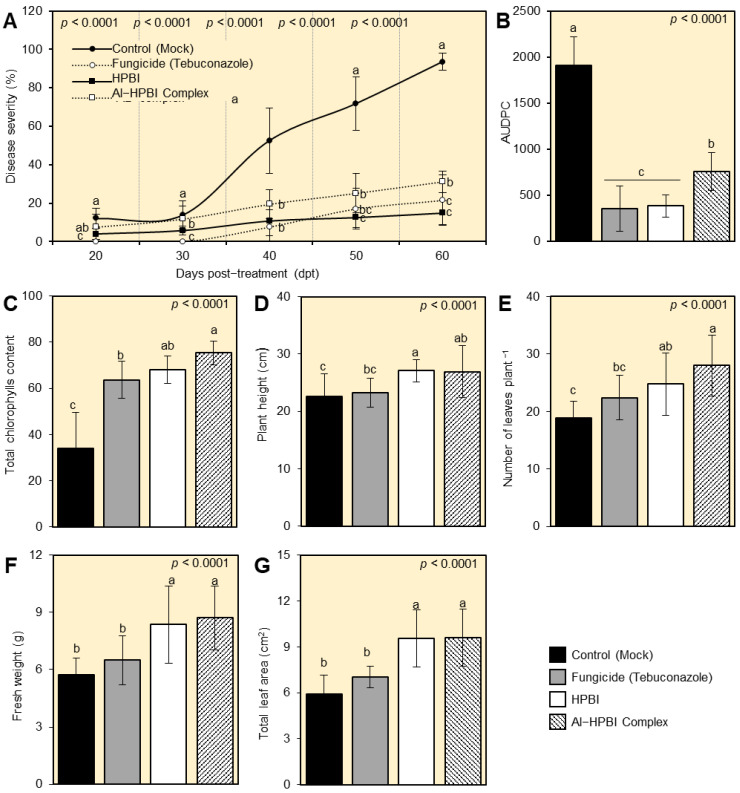
HPBI and its aluminum complex suppress the progression of Fusarium wilt disease and enhance the growth of infected pepper plants under greenhouse conditions. (**A**) Effect of HPBI and its aluminum complex on disease severity (%) at 20-, 30-, 40-, 50-, and 60-days post-treatment (dpt). (**B**) Area under disease progress curve (AUDPC). (**C**) Total chlorophylls content. (**D**) Plant height (cm). (**E**) Number of leaves plant^−1^. (**F**) Fresh weight (g). (**G**) Total leaf area (cm^2^). Values represent the means ± standard deviation (means ± SD) of six biological replicates (*n* = 6). Different letters signify statistically significant differences between treatments (*p* < 0.05).

Generally, HPBI and its aluminum complex significantly reduced the symptoms development of Fusarium wilt disease on treated pepper plants compared with the mock-treated infected plants (Figure 3A). Although mock-treated pepper plants showed a progressive surge in terms of disease severity (%), root treatment with HPBI and Al−HPBI complex significantly reduced the percentage of disease severity at 20, 30, 40, 50, and 60 dpt, and till the end of the experiment (Figure 3B). Regardless of the commercial fungicide, HPBI was more effective than its aluminum complex and had lower disease severity (%), particularly at early stages (20 and 30 dpt), and till 60 dpt (14.88 ± 6.24%; Figure 3A). Similarly, both HPBI and its aluminum complex significantly lessened the area under the disease progress curve (AUDPC) compared with the mock-treated control (Figure 3B). Among all treatments, the commercial fungicide ‘Hattrick’ showed the lowest AUDPC (354.86 ± 245.03), followed by HPBI (382.44 ± 120.74), which were not significantly different from each other. On the other hand, the mock-treated pepper plants had the highest AUDPC (1910.18 ± 306.803) at 60 dpt.

### 3.3. HPBI and Its Aluminum Complex Enhance the Growth Variables of Infected Pepper Plants

At 60 dpt, root treatment with HPBI and its aluminum complex significantly enhanced all studied growth parameters, including total chlorophyll (SPAD; Figure 3C), plant height (Figure 3D), the number of leaves per plant (Figure 3E), fresh weight (Figure 3F), and total leaf area (Figure 3G) with significant differences between them but significantly higher than the mock-treated control. Taken together, these findings suggest that root treatment with HPBI and its aluminum complex has no phytotoxicity on the treated pepper plants.

### 3.4. HPBI and Its Aluminum Complex Mitigate the Oxidative Stress of F. equiseti-Infected Plants

**Figure 4:**
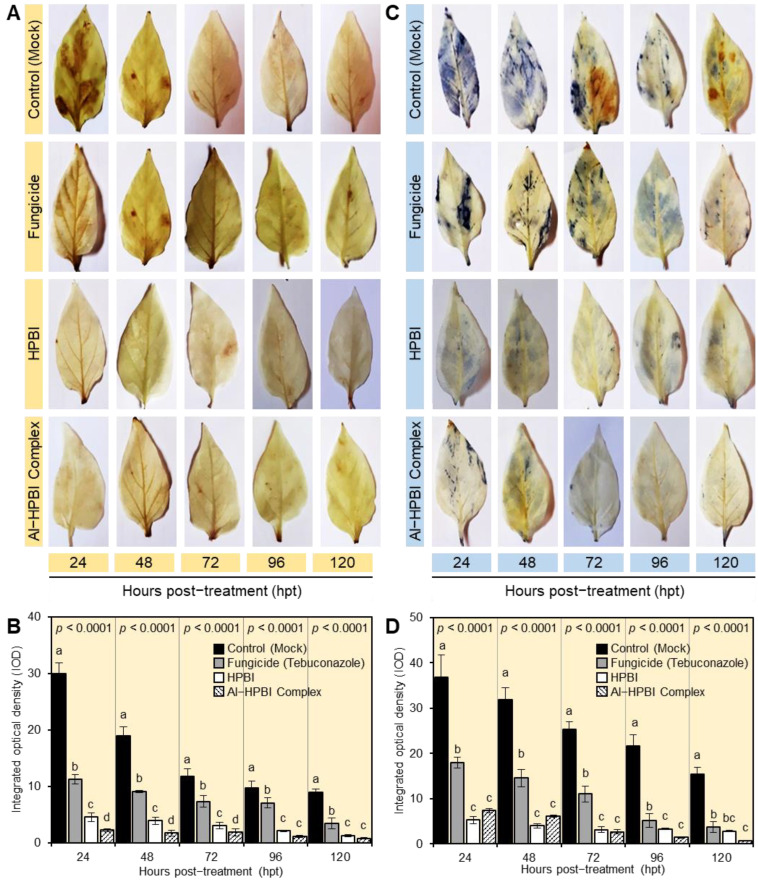
HPBI and its aluminum complex relieve oxidative stress in *Fusarium equiseti-infected* pepper plants under greenhouse conditions. (**A**) In situ histochemical visualization of hydrogen peroxide (H_2_O_2_) at 72 hpt with HPBI or its aluminum complex. (**B**) Integrated optical density (IOD) of H_2_O_2_ at 72 hpt with HPBI or its aluminum complex. (**C**) In situ histochemical localization of superoxide anion (O_2_^•−^) at 72 hpt with HPBI or its aluminum complex. (**D**) Integrated optical density (IOD) of O_2_^•−^ at 72 hpt with HPBI or its aluminum complex. Bars represent the means ± standard deviation (means ± SD) of six biological replicates (*n* = 6). Different letters signify statistically significant differences between treatments (*p* < 0.05).

DAB-based in situ visualization of H_2_O_2_ (Figure 4A) showed that leaves from mock-treated *F*. *equiseti-*infected pepper plants accumulate a higher H_2_O_2_ content than other treatments at 24, 48, 72, 96, and 120 hpt. However, root treatment with HPBI and Al−HPBI complex markedly decreased the H_2_O_2_ levels within the *F*. *equiseti*-infected plants with the superiority of HPBI over its aluminum complex (Figure 4B). Similarly, NBT-based in situ histochemical localization of O_2_^•−^ suggested that *F*. *equiseti*-infected plants accumulate higher O_2_^•−^ levels in the leaves (Figure 4C). Nevertheless, root treatment with HPBI or its aluminum complex significantly reduced the O_2_^•−^ content within infected plants with a greater effect of HPBI (Figure 4D).

### 3.5. HPBI and Al−HPBI Complex Induce the Antioxidant Defense Machinery of F. equiseti-Infected Plants

**Figure 5:**
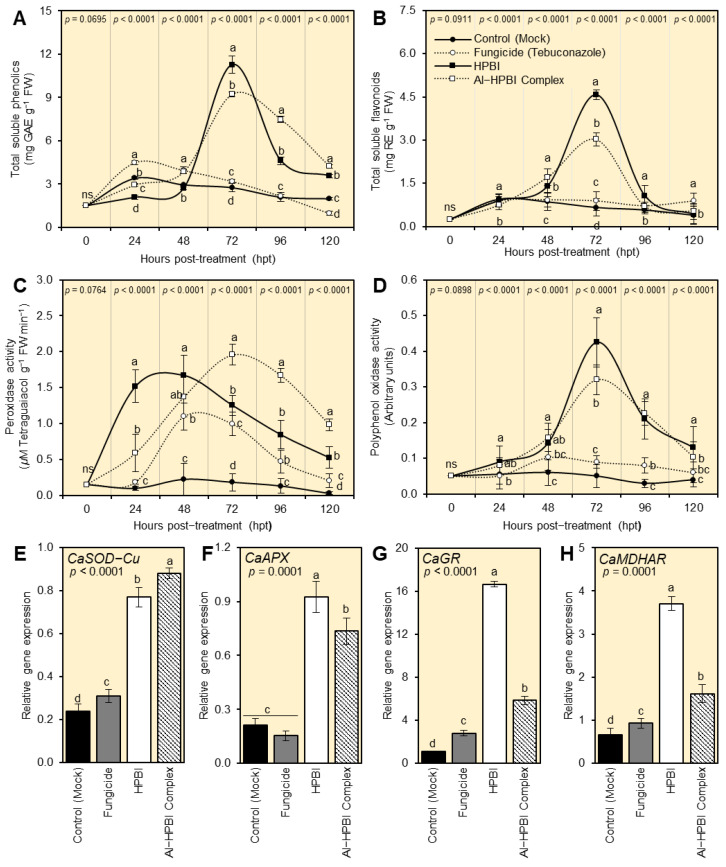
HPBI and its aluminum complex induce the antioxidant defense system of *Fusarium equiseti-*infected pepper plants under greenhouse conditions. (**A**) Total soluble phenolics (mg GAE g^−1^ FW). (**B**) Total soluble flavonoids (mg RE g^−1^ FW). (**C**) Peroxidase activity (μM Tetraguaiacol g^−1^ FW min^−1^). (**D**) Polyphenol oxidase activity (arbitrary units). (**E**–**H**) Relative gene expression of superoxide dismutase [Cu-Zn] (*CaSOD-Cu*), L-ascorbate peroxidase 1, cytosolic (*CaAPX*), glutathione reductase, chloroplastic (*CaGR*), and monodehydroascorbate reductase (*CaMDHAR*). Values represent the means ± standard deviation (means ± SD) of six biological replicates (*n* = 6). Different letters signify statistically significant differences between treatments (*p* < 0.05).

To better understand the physiological and biochemical mechanisms of HPBI and its aluminum complex within *F. equiseti*-infected plants, non-enzymatic and enzymatic antioxidant defense machinery were further investigated. Briefly, root treatment with HPBI or Al−HPBI complex significantly enhanced the non-enzymatic antioxidant defense machinery as expressed by total soluble phenolics (Figure 5A) and flavonoids (Figure 5B). Moreover, both compounds improved the enzymatic antioxidants as expressed by the enzymatic activities of guaiacol-dependent peroxidases (POX; Figure 5C) and polyphenol oxidase (PPO; Figure 5D), and the gene expression of superoxide dismutase [Cu-Zn] (*CaSOD-Cu*; Figure 5E), L-ascorbate peroxidase 1, cytosolic (*CaAPX*; Figure 5F), glutathione reductase, chloroplastic (*CaGR*; Figure 5G), and monodehydroascorbate reductase (*CaMDHAR*; Figure 5H).

#### 3.5.1. HPBI and Al−HPBI Complex Boost the Content of Total Soluble Phenolics and Flavonoids of *F. equiseti*-Infected Pepper Plants

Both tested compounds (HPBI and Al−HPBI complex) boosted the content of total soluble phenolics of *F. equiseti*-infected pepper plants (Figure 5A). It is worth mentioning that the application of HPBI induced the accumulation of total soluble phenolics within treated pepper plants to reach its highest peak at 72 hpt (11.27 ± 0.58 mg GAE g^−1^ FW), followed by Al−HPBI complex (9.22 ± 0.17 mg GAE g^−1^ FW) at the same time point. Likewise, the application of HPBI and its aluminum complex augmented the total soluble flavonoid content of *F. equiseti*-infected pepper plants (Figure 5B). The total soluble flavonoid content dramatically increased to reach its highest peak at 72 hpt when pepper plants were treated with HPBI (4.57 ± 0.17 mg RE g^−1^ FW), followed by the Al−HPBI complex (3.02 ± 0.23 mg RE g^−1^ FW), but it dropped thereafter when measured at 96 and 120 hpt (Figure 5B).

#### 3.5.2. HPBI and Al−HPBI Complex Induce Antioxidant-Related Enzymes of *F. equiseti*-Infected Pepper Plants

Generally, the enzymatic activities of POX (Figure 5C) and PPO (Figure 5D) fluctuated after the root application of HPBI and its aluminum complex. It is worth mentioning that HPBI induced the activity of POX earlier than PPO and faster than the Al−HPBI complex. Briefly, the enzymatic activity of POX dramatically increased at 24 hpt with HPBI (1.52 ± 0.22 μM tetraguaiacol g^−1^ FW min^−1^), and continued to increase slightly to reach its highest peak at 48 hpt (1.67 ± 0.28 μM tetraguaiacol g^−1^ FW min^−1^); however, the Al−HPBI complex hit its highest peak at 72 hpt to record the highest POX activity (1.96 ± 0.14 μM Tetraguaiacol g^−1^ FW min^−1^) among all treatments (Figure 5C). On the other hand, the enzymatic activity of PPO did not change noticeably during the first two days post-treatment with HPBI or its aluminum complex; nevertheless, it dramatically increased at 72 hpt with HPBI (0.42 ± 0.07 arbitrary units) and Al−HPBI complex (0.32 ± 0.04 arbitrary units), with superiority for HPBI (Figure 5D).

Furthermore, our study showed that HPBI and its aluminum complex significantly enhanced the transcript levels of four antioxidant-related genes including *CaSOD-Cu* (Figure 5E), *CaAPX* (Figure 5F), *CaGR* (Figure 5G), and *CaMDHAR* (Figure 5H). Although the Al−HPBI complex greatly upregulated the *CaSOD-Cu* gene more than HPBI, the expression levels of the other three genes (*CaAPX*, *CaGR*, and *CaMDHAR*) were significantly increased upon the treatment with HPBI.

### 3.6. F. equiseti Genome Possesses a Putative Sterol 24-C-methyltransferase (EGR6)

To better understand the molecular mechanisms behind the antifungal activity of HPBI and its aluminum complex, we in silico analyzed its ability to inhibit the synthesis of sterols (main components for the formation of fungal cell walls) via the inhibition of Sterol 24-C-methyltransferase (*EGR6*). However, because the *EGR6* gene from *F. equiseti* is not cloned yet, and is not well identified, we carried out a comparative in silico analysis to identify the putative *ERG6* gene(s) from *F. equiseti.* The amino acid (AA) sequence of *EGR6* from *F. oxysporum* NRRL 32931 (henceforth *FoEGR6*; GenBank accession no. XP_031038350.1; 382 aa) was used as a query sequence to identify putative *EGR6* candidates from *F. equiseti*.

Briefly, in silico analysis using the protein–protein BLAST (BLASTp) tool showed that the *F. equiseti* genome possesses six predicted “Unnamed Protein Product” sequences (Table S1) that produce significant similarities to *FoEGR6* from *F. oxysporum* NRRL 32931. Moreover, the multiple protein sequence alignment using the Constraint Based Alignment tool (COBALT) showed that all predicted sequences have relatively high homology with *FoEGR6* (Supplementary Figure S1A). However, only one protein from *F. equiseti* fully covered the query sequence of *FoEGR6* from *F. oxysporum* NRRL (Query Cover = 100%) and has a relatively high homology with it (Identity = 95.03%) with a low E. value (0.0). This protein was an “Unnamed Protein Product” (henceforth *FeEGR6*; GenBank accession no. CAG7563035.1; 381 aa) (Supplementary Table S1). Therefore, we focused on this protein for further in silico analysis.

**Figure 6:**
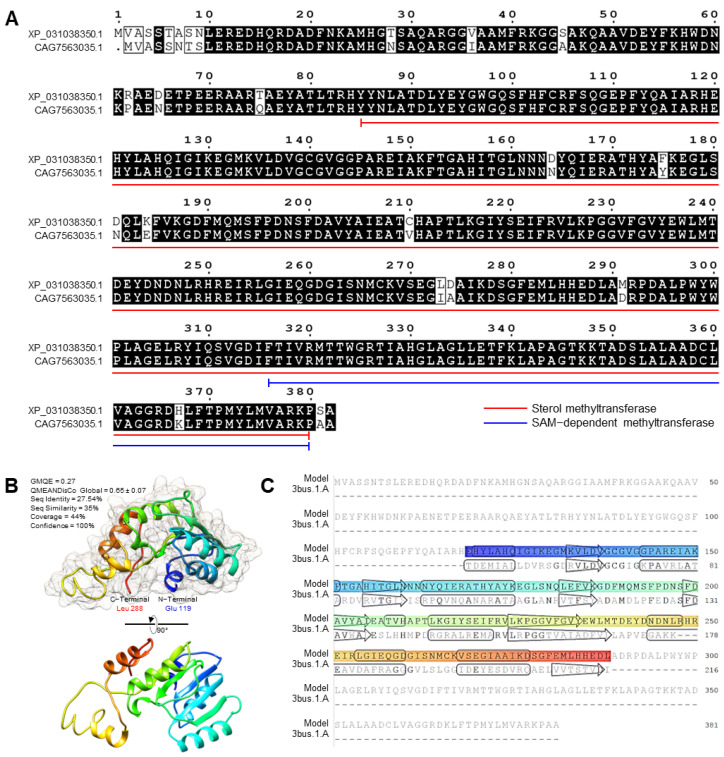
In silico analysis of Sterol 24-C-methyltransferase (*EGR6*) of *Fusarium equiseti*. (**A**) Multiple sequence alignments of *EGR6* from *F. oxysporum* (*FoEGR6*; XP_031038350.1) and *F. equiseti* (*FeEGR6*; CAG7563035.1). Black shading denotes conserved amino acids, while white background denotes a low similarity score. Red and blue lines below the sequences indicate the conserved domains of Sterol methyltransferase and SAM−dependent methyltransferase, respectively. Whiskers suggest the beginning and the ending of each domain. (**B**) The predicted crystallographic three-dimensional (3D) modeling of 24-C-methyltransferase from *F. equiseti* (*FeEGR6*; CAG7563035.1). (**C**) Model–template alignment of *FeEGR6* from *F. equiseti*. GMQE: Global model quality.

The AA sequence of putative *FeEGR6* was aligned with the AA sequences of *FoEGR6* (Figure 6A). The AA alignment showed high similarity and conserved sequences in both Sterol methyltransferase and SAM−dependent methyltransferase domains (Figure 6A). Functional analysis and interactively prediction of conserved domains and important sites of putative *FeEGR6*, as well as *FoEGR6*, was done using the InterPro Scan tool. Our findings suggest a high topological similarity between both proteins (*FeEGR6* and *FoEGR6*) (Supplementary Figure S1B). Briefly, both proteins possess three conserved domains including Sterol methyltransferase C−terminal (IPR013705), SAM-dependent methyltransferase SMT-type (IPR030384), and Methyltransferase type 11 (IPR013216) (Supplementary Figure S1B). Moreover, both proteins have a SAM-dependent methyltransferase superfamily (IPR029063).

The crystallographic three-dimensional (3D) structure of *FoEGR6* and *FeEGR6* was predicted using the crystal structure of 4’-O-methyltransferase (*RebM*; Protein Data Bank [PDB ID]: 3BUS.1.A), and refined to 2.63 Å resolution with excellent alignment statistics (Figure 6B and Figure S2A). Briefly, about 48% (residues Ile 116 to Leu 302) of *FoEGR6* have been modeled with the template protein (seq identity = 26.09%, seq similarity = 34%, and confidence = 100%) with accepted global model quality estimation (GMQE = 0.29) and good absolute quality (QMEANDisCo Global = 0.60 ± 0.06) (Supplementary Figure S2A). The predicted 3D model of *FoEGR6* is a monomer composed of 8 *α*-helices and 5 *β*-sheets (Supplementary Figure S2A,B) with considerable predicted local similarity to the target.

Likewise, approximately 44% (residues Glu 119 50 to Leu 288) of *FeEGR6* have been modeled with the target protein (seq identity = 27.54%, seq similarity = 35%, and confidence = 100%) with accepted GMQE (0.27) and good QMEANDisCo Global = 0.65 ± 0.07 (Figure 6B). Like *FoEGR6,* the predicted model of *FeEGR6* contains eight *α*-helix ribbons and five stranded *β*-wings (Figure 6B,C) with considerable predicted local similarity to the target.

### 3.7. Molecular Docking Analysis Reveals Ligand–Protein Interactions

**Figure 7:**
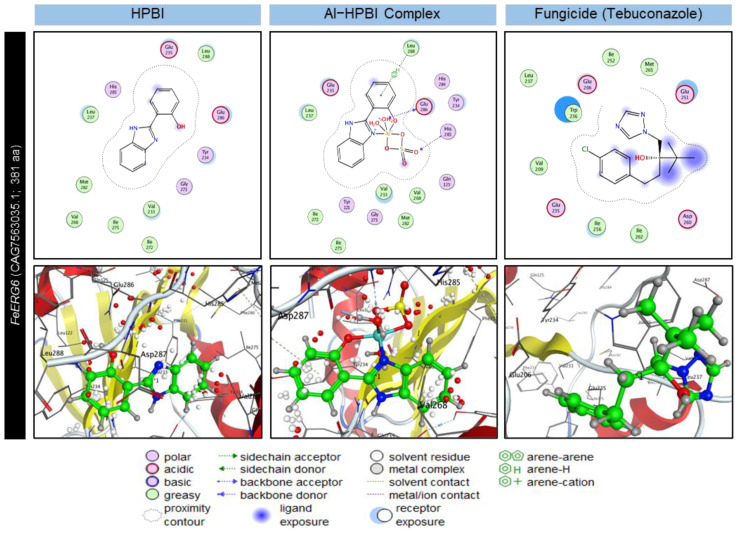
Two-dimensional (2D) and three-dimensional (3D) docking interaction of HPBI, Al−HPBI complex, and Fungicide (Tebuconazole) with 24-C-methyltransferase (*ERG6*) from *F. equiseti* (*FeEGR6*; CAG7563035.1).

To better understand the mode of action of HPBI and its aluminum complex against *F. equiseti*, we computationally investigated their ability to bind the active site(s) of the 3D structure of *FoEGR6* and *FeEGR6* proteins (Figure 7 and Figure S3). Molecular docking analysis showed that the free ligand (HPBI) formed one arene H-bond with the residue Leu 238 to interact with *FoEGR6* (Supplementary Figure S3) while with *FeEGR6* only electrostatic attraction in all sites was noticed (Figure 7). It is worth mentioning that HPBI revealed a low binding energy score when docked with *FoEGR6* and *FeEGR6* proteins (S = −5.38 and −5.36 Kcal·mol^−1^; respectively) and high root-mean-square deviations of atomic locations (RMSD = 2.50 and 1.94 Å; respectively) (Table 1). On the other hand, the Al−HPBI complex formed five bonds with the amino acid residues of *FoEGR6*; the first is the side chain acceptor with Gln 128, the backbone acceptor with the His 286, the backbone donor with Glu 236 and Glu 287, and finally, the arene H bond with Leu 289 (Supplementary Figure S3). However, the Al−HPBI complex formed only three bonds besides the electrostatic interaction with most sites of the *FeEGR6* protein (Figure 7). These results indicate that the complex revealed a high binding energy score when docked with *FoEGR6* and *FeEGR6* proteins (Docking scores = −5.56 and −6.51 Kcal·mol^−1^; respectively) and low root-mean-square deviations of atomic locations (RMSD = 1.5986 and 1.6471 Å; respectively) (Table 1). If the results of the complex and legend are compared to the fungicide (Hattrick), we find that the complex is better interconnected than the fungicide and legend in the case of the *FeEGR6* protein. However, in terms of interaction with *FoEGR6* protein, the fungicide was slightly stronger than the complex this is due to the formation of a backbone donor and acceptor stronger than in the complex. From the previous results, and depending on what was obtained in practice, it can be recommended to use ligand its complex as a safe alternative to the fungicide with this fungus.

## 4. Discussion 

In the current study, we proposed two potential benzimidazole derivatives, including 2-(2-hydroxyphenyl)-1H-benzimidazole (HPBI) and its aluminum complex (Al−HPBI complex), as alternatives to chemical fungicides against Fusarium wilt disease. Benzimidazole derivatives are a group of chemical compounds that are widely used in different fields, and it is necessary to research their biological importance [57]. Our in vitro investigation showed that both compounds (HPBI and Al−HPBI complex) revealed potent dose-dependent antifungal activity against *F. equiseti* and HPBI completely suppressed the linear mycelial growth of *F. equiseti* when treated with 2 mM, which was similar to “Hattrick” fungicide at its recommended dose (1 cm^3^ L^−1^). Although previously we proved that metal complexation of a ligand might enhance its efficacy against phytopathogenic fungi, such as *F. equiseti* [5], our in vitro studies showed that the application of the ligand itself (HPBI) was slightly better than its aluminum complex.

We believe that the kind of metal ion might affect the efficacy of the ligand. For instance, ligand complexation with ruthenium III significantly enhanced the efficacy of 3-(4-dimethyl amino-phenyl)-1-{6-[3-(4 dimethyl amino-phenyl)-a cryloyl]-pyridin-2-yl}-propanone (DMAPAPP) [5]; however, complexation with aluminum slightly reduced the efficacy of HPBI. This is probably due to the large molecular weight of the Al−HPBI complex, which reduces its penetration into the membrane of fungal cells. In the current study, the molecular weight of the Al−HPBI complex was 368.30 g.mol^−1^, while the molecular weight of HPBI was around 210.24 g.mol^−1^. It was reported previously that benzimidazole derivatives with a molecular weight less than 300 g.mol^−1^ were more effective against phytopathogenic fungi than those with a high molecular weight [24].

The antifungal activity of HPBI and its aluminum complex might be due to their ability to interact with *F. equiseti* proteins, particularly sterols biosynthesis-related proteins such as Sterol 24-C-methyltransferase (*EGR6*). Sterols, such as ergosterol, are essential components for the formation of fungal cell walls. However, the *EGR6* gene from *F. equiseti* is not cloned yet and is even poorly identified. We carried out a comparative in silico analysis to identify the putative *ERG6* gene(s) from *F. equiseti.* Our *in-silico* analysis showed that the *F. equiseti* genome possesses six predicted “Unnamed Protein Product” sequences that produce significant similarities to *FoEGR6* from *F. oxysporum* NRRL 32931. However, only one of these six proteins from *F. equiseti* was fully covered the query sequence of *FoEGR6* from *F. oxysporum* NRRL, which was the “Unnamed Protein Product” (*FeEGR6*; GenBank accession no. CAG7563035.1; 381 aa). The homology between sterol methyltransferases (SMT) from plants and ERG6 from fungi was reported previously [58].

Functional analysis and interactively prediction of conserved domains and important sites of putative *FeEGR6*, as well as *FoEGR6*, showed that both proteins have a Sterol methyltransferase domain that was found the C-terminus, which was previously reported to be associated with steroid biosynthesis in fungi and plants [59]. Moreover, both proteins (*FeEGR6* and *FoEGR6*) have a SAM−dependent methyltransferase SMT−type, which is a characteristic homologous superfamily in ERG6 proteins and is implicated in the biosynthesis of ergosterol, which is critical for plasma membrane formation and performance [60]. Our molecular docking studies showed that HPBI and its aluminum complex strongly interact with the *FeEGR6* from *F. equiseti* as well as *FoEGR6* from *F. oxysporum.*

Briefly*,* the Al−HPBI complex formed five bonds with the amino acid residues of the *FoEGR6* protein. However, it formed only three bonds with *FeEGR6* from *F. equiseti*, in addition to its electrostatic contact with the majority of sites. On the other hand, HPBI formed only one arene H-bond with Leu 238 amino acid residue in the *FoEGR6* protein, while strong electrostatic attractions in all sites were observed with *FeEGR6*. It is worth mentioning that the Al−HPBI complex showed a better interconnection with *FeEGR6* protein than the fungicide (Hattrick), which was slightly stronger when interconnected with *FoEGR6* protein from *F. oxysporum.* However, further studies are required to clone this protein and experimentally confirm its biological function.

Although molecular docking was used as a quick tool for virtual screening and compound optimization, it may produce unreliable binding affinity predictions [61,62]. In other words, docking performance is repeatedly of low quality with no consensus reproducibility among various docking tools [61]. Moreover, predicted binding affinities might be inaccurate, regardless of the appropriately predicted binding pose [62]. H-bonding and the water description are the main issues in predicting binding affinity via docking [61,62]. In the current study, although all the water molecules around the protein were eliminated and hydrogen was added, using docking as a stand-alone approach is questionable and required further analysis. For further studies, we recommend using comprehensive approaches such as quantum-mechanical (QM)-based methods [63], dynamic docking [64], and molecular dynamics (MD) simulations [65] to identify more reliable binding poses and to validate the results of the classical docking. Finally, the most promising poses should be further analyzed using short MD simulations (usually 200 ns), followed by WaterMap analysis.

Furthermore, our greenhouse findings showed that both compounds (HPBI and Al−HPBI complex) significantly reduced the development of Fusarium wilt disease on pepper plants, as indicated by reduced disease severity and lower AUDPC compared with non-treated plants. This might be due to their strong antifungal activity, as mentioned above, or it might be due to the activation of the plant defense system. The antimicrobial activities of benzimidazole derivatives have been demonstrated previously against several pathogenic bacteria, such as *Staphylococcus* sp., *Enterococcus* sp., *Escherichia* sp., *Enterobacter* sp., and *Klebsiella* sp. [24], as well as phytopathogenic fungi, such as *Botrytis cinerea*, *Alternaria solani*, *Cytospora* sp., *Colletotrichum gloeosporioides*, *Fusarium culmorum*, and *F. solani* [24,66]. The antimicrobial activity of benzimidazole derivatives was reported previously to be associated with the length of the alkyl chain [67]; however, the effect of metal complexation of benzimidazole derivatives is poorly understood. Moreover, benzimidazoles might affect the membrane permeability and cell wall function of phytopathogenic fungi [24]. This supports our hypothesis that HPBI and Al−HPBI complex interact with *EGR6* proteins to block the sterols biosynthesis, probably via sterol 24-C-methyltransferase inhibition, causing secondary degenerative changes in the fungal cell wall.

It is worth mentioning that although root application of both HPBI and Al−HPBI complex significantly reduced the severity of Fusarium wilt disease compared with the non-treated plants, both compounds did not show any phytotoxicity on treated plants, as revealed by enhanced growth performance (total chlorophyll content, plant height, number of leaves per plant, fresh weight, and total leaf area). This might be due to the direct antioxidant properties of both compounds or the activation of antioxidant defense machinery. The antioxidant capacity and free radical scavenging properties of benzimidazole derivatives were confirmed previously [68,69] and were reported to be associated with their structural features [70]. Taken together, we believe that treating pepper plants with HPBI or Al−HPBI complex might reduce the development of Fusarium wilt disease in pepper plants by directly increasing the antioxidant capacity within infected plants.

Although the infection with *F. equiseti* noticeably increased the reactive oxygen species (ROS) in non-treated pepper plants as expressed by higher levels of hydrogen peroxide (H_2_O_2_) and superoxide anion (O_2_^•−^), root application of HPBI or its aluminum complex significantly suppressed this oxidative stress via the induction of a complex multilayered antioxidant defense system. This system involves two main mechanisms: (I) enzymatic antioxidants and (II) nonenzymatic antioxidants [71]. Enzymatic antioxidants work as the first defense line against ROS [72,73,74,75], whereas nonenzymatic antioxidants work as the second defense line [75].

In the enzymatic antioxidant defense machinery, superoxide dismutase (SOD) catalyzes the conversion of superoxide ion (O_2_^−•^) to hydrogen peroxide (H_2_O_2_) [76], then ascorbate peroxidase (APX) catalyzes the transformation of H_2_O_2_ into H_2_O. Moreover, peroxidase (POX) controls the level of H_2_O_2_ in plant tissues [72] and directly scavenges H_2_O_2_ and O_2_^−•^ to reduce their reactivity [73]. Our findings showed that the root application of HPBI or its aluminum complex significantly enhanced the enzymatic activities of POX and PPO and upregulated four antioxidant-related genes including *CaSOD-Cu*, *CaAPX*, *CaGR*, and *CaMDHAR* at 72 hpt. Collectively, these findings support our suggestion that treatment with HPBI or its aluminum complex induces the activation of enzymatic antioxidant defense machinery within infected pepper plants.

In addition to enzymatic antioxidant defense machinery, the root application of HPBI and Al−HPBI complex play a key role in the activation of non-enzymatic antioxidant defense machinery. Non-enzymatic antioxidants include phenolics and flavonoids, as well as lipophilic antioxidants such as carotenoids [75]. Our findings showed that root application of HPBI or its aluminum complex significantly enhanced the endogenous content of total soluble phenolics and flavonoids in pepper leaves. These findings suggest that HPBI and Al−HPBI complex stimulate the activation of a multilayered antioxidative system in infected plants to mitigate the harmful effects of ROS and preserve their homeostasis.

## 5. Conclusions

In the current study, we propose two benzimidazole derivatives (HPBI and Al−HPBI complex) that can successfully suppress the soil-borne phytopathogenic fungus, *F. equiseti*, the causal agent of Fusarium wilt disease in pepper plants. Both compounds showed strong dose-dependent antifungal activity against *F. equiseti*. This antifungal activity may be due to their ability to interact with *ERG6* protein. Moreover, root application of both compounds noticeably reduced the disease symptoms in infected pepper plants, with no phytotoxicity symptoms as expressed by normal growth performance. According to molecular docking predictions, this antifungal activity may be due to their capacity to interact with *ERG6* protein. Finally, treatment with HPBI and Al−HPBI complex promotes the activation of both enzymatic (POX, PPO, SOD, APX, GR, and MDHAR) and non-enzymatic (total phenolics and flavonoids) antioxidant defense machinery. Collectively, the findings of the current study suggest the potential application of HPBI and its aluminum complex as a novel control alternative against *F. equiseti* and interpret the physiological, biochemical, and molecular mechanisms behind their protective role.

## Figures and Tables

**Table 1:** Docking score, RMSD-refine, and strain energy of HPBI, Al−HPBI complex, and Hattrick Fungicide with *FeEGR6* and *FoEGR6* proteins.

Compound	Docking Scores (S) (Kcal·mol^−1^)		RMSD-Refine (Å)		Strain Energy (Kcal·mol^−1^)	
	*FoEGR6*	*FeEGR6*	*FoEGR6*	*FeEGR6*	*FoEGR6*	*FeEGR6*
HPBI	−5.38	−5.36	2.50	1.94	39.06	39.49
Al−HPBI Complex	−5.56	−6.51	1.60	1.65	40.23	40.50
Hattrick Fungicide	−6.64	−6.05	1.32	3.15	39.56	39.60

RMSD: root-mean-square deviations of atomic locations.

## Data Availability

The datasets generated and/or analyzed during the current study are available from the corresponding author upon reasonable request.

## Supplementary Materials

The following supporting information can be downloaded at: https://www.mdpi.com/article/10.3390/jof9020244/s1, Table S1: Sequences from *Fusarium equiseti* that produce significant Alignment with sterol 24-C-methyltransferase (*ERG6*) gene from *Fusarium oxysporum* NRRL 32931; Figure S1: *In silico* analysis of Sterol 24-C-methyltransferase (*EGR6*) of *Fusarium equiseti*; Figure S2: The crystallographic three-dimensional (3D) modeling of 24-C-methyltransferase from *F. oxysporum* (*FoEGR6*); Figure S3: Two-dimensional (2D) and three-dimensional (3D) docking interaction of HPBI, Al−HPBI Complex, and Fungicide (Tebuconazole) with 24-C-methyltransferase from *F. oxysporum* (*FoEGR6*; XP_031038350.1).
