# Supplementary material for: Benzimidazole Derivatives Suppress Fusarium Wilt Disease via Interaction with ERG6 of Fusarium equiseti and Activation of the Antioxidant Defense System of Pepper Plants
Source: J Fungi (Basel). 2023 Feb 12;9(2):244. doi: 10.3390/jof9020244 (PMC9961032; doi:10.3390/jof9020244)
Supplement: Supplementary file 1 [file jof-09-00244-s001.zip › jof-2170836-supplementary.pdf]

Journal of Fungi

MDPI

Supplementary materials

Benzimidazole Derivatives Suppress Fusarium Wilt Disease via Interaction with *ERG6* of *Fusarium equiseti* and Activation of the Antioxidant Defense System of Pepper Plants

Asmaa El-Nagar [1,†], Abdelnaser A. Elzaawely [1], Hassan M. El-Zahaby [1], Tran Dang Xuan [2,3], Tran Dang Khanh [4,5], Mohamed Gaber [6], Nadia El-Wakeil [6], Yusif El-Sayed [6] and Yasser Nehela [1,*,†]

1 Department of Agricultural Botany, Faculty of Agriculture, Tanta University, Tanta 31527, Egypt
2 Transdisciplinary Science and Engineering Program, Graduate School of Advanced Science and Engineering, Hiroshima University, Hiroshima 739-8529, Japan
3 Center for the Planetary Health and Innovation Science (PHIS), The IDEC Institute, Hiroshima University, Hiroshima 739-8529, Japan
4 Agricultural Genetic Institute, Pham Van Dong Street, Hanoi 122000, Vietnam
5 Center for Agricultural Innovation, Vietnam National University of Agriculture, Hanoi 131000, Vietnam
6 Chemistry Department, Faculty of Science, Tanta University, Tanta 31527, Egypt
* Correspondence: yasser.nehela@ufl.edu
† These authors contributed equally to this work.

Journal of Fungi MDPI

Supplementary Table S1. Sequences from *Fusarium equiseti* that produce significant Alignment with sterol 24-C-methyltransferase (*ERG6*) gene from *Fusarium oxysporum* NRRL 32931 [a].

**Gene** Description	Protein				Protein-Protein Alignment statistics				
	GenBank Accession	aa	Theoretical isoelectric point (pI)	Molecular weight (MW)	Max Score	Total Score	Query Cover (%)	Identity (%)	E value
Unnamed Protein Product	CAG7563035.1	381	5.93	42536.94	715	715	100	95.03	0.0
Unnamed Protein Product	CAG7563809.1	381	5.57	43083.56	498	498	96	67.12	1e-177
Unnamed Protein Product	CAG7563160.1	345	5.55	38171.43	76.6	76.6	52	27.98	9e-16
Unnamed Protein Product	CAG7562504.1	279	6.22	30823.11	64.7	64.7	27	32.38	5e-12
Unnamed Protein Product	CAG7562905.1	521	6.06	59867.38	47.0	47.0	46	22.16	6e-06
Unnamed Protein Product	CAG7563468.1	246	5.49	27835.68	39.7	39.7	26	22.12	7e-04

[a] The listed putative gene candidates were identified using the protein-protein BLAST (BLASTp) using sterol 24-C-methyltransferase (*FoEGR6*; GenBank accession no. XP_031038350.1; 382 aa) from *Fusarium oxysporum* NRRL 32931 as a query sequence against *F. equiseti* genome available in GenBank, national center for biotechnology information website (NCBI, http://www.ncbi.nlm.nih.gov/gene/), using the compositionally adjusted substitution matrices [44,57].

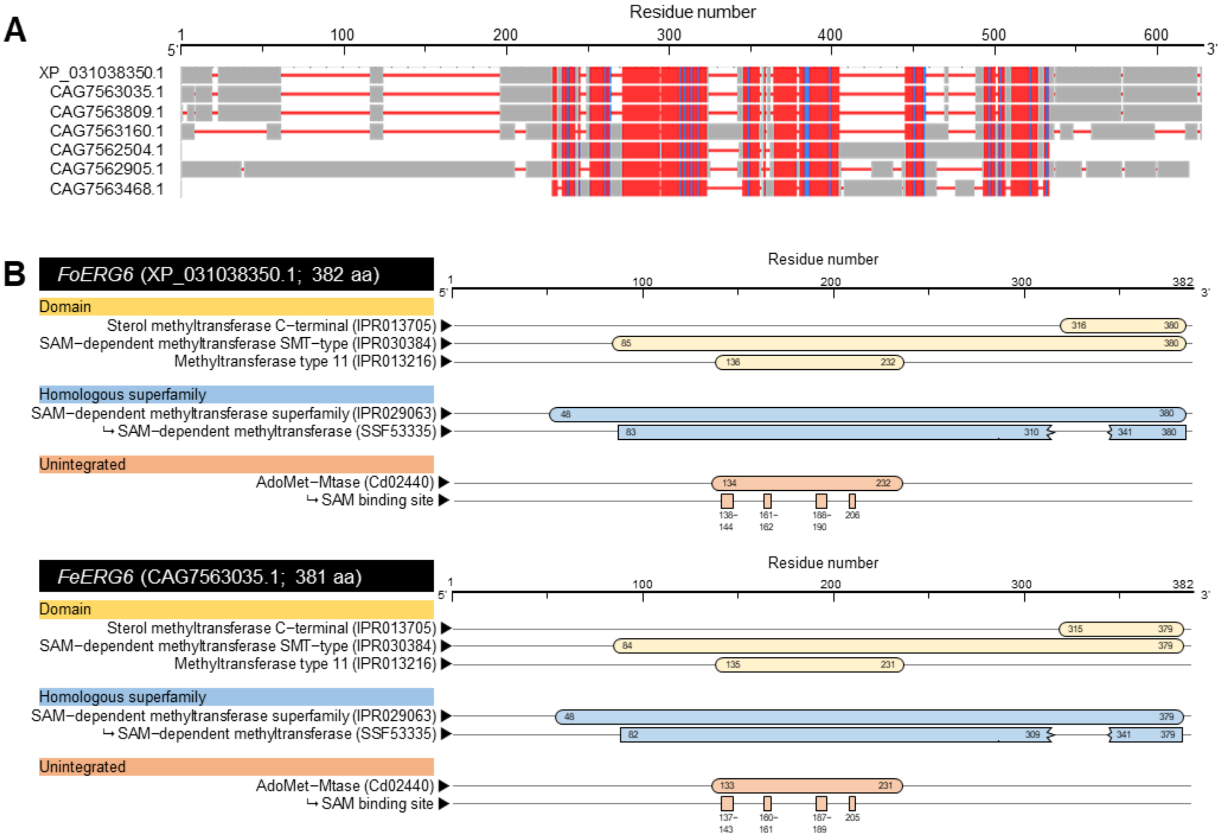

Supplementary Figure S1. ***In silico*** **analysis of Sterol 24-C-methyltransferase (*EGR6*) of *Fusarium equiseti*.** (**A**) Multiple protein sequence alignments of *EGR6* from *F. oxysporum* and its homologs from *F. equiseti* using the Constraint-Based Alignment tool (COBALT). (**B**) The protein functional and conserved domains analysis of *FoEGR6* (XP_031038350.1) and *FeEGR6* (CAG7563035.1) using the InterPro Scan tool.

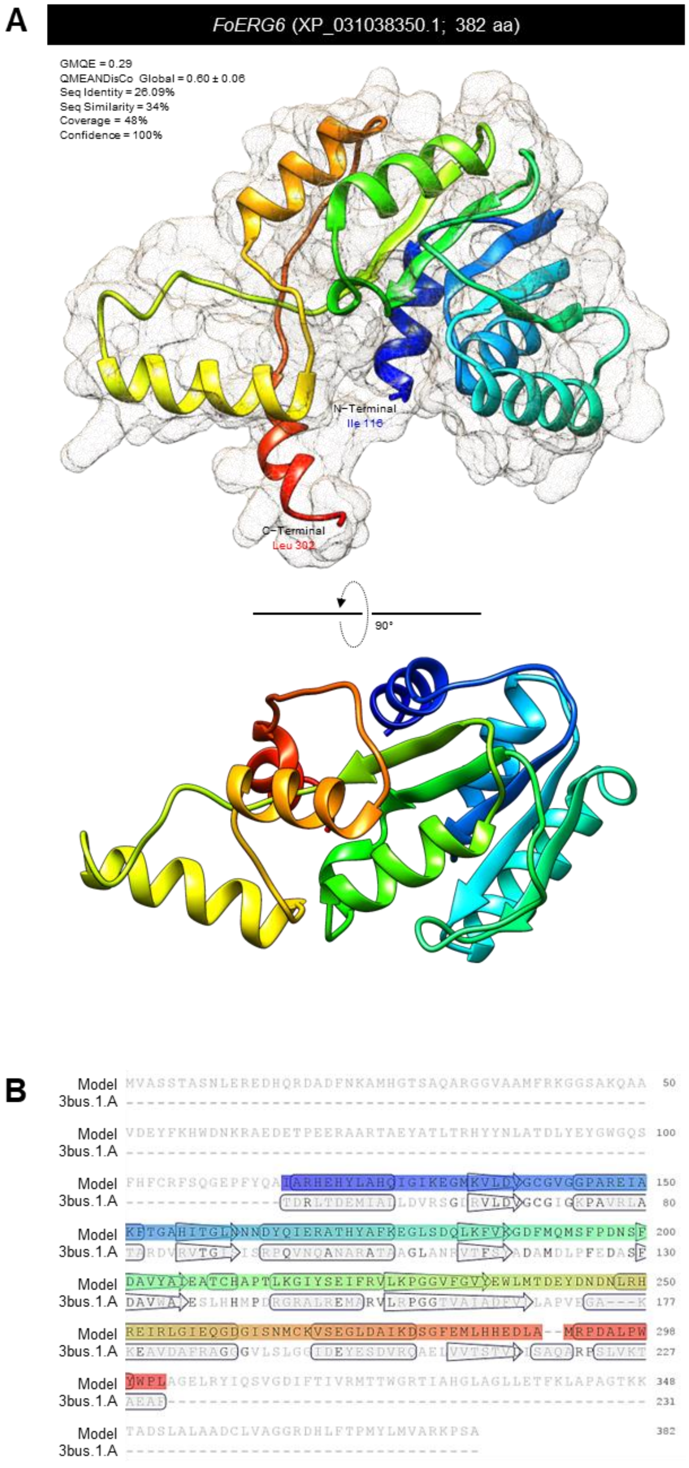

Supplementary Figure S2. The crystallographic three-dimensional (3D) modeling of 24-C-methyltransferase from *F. oxysporum* (*FoEGR6*). (**A**) The predicted 3D structure model and its associated mesh surface of *FoEGR6* (XP_031038350.1) from *F. oxysporum*. (**B**) Model–template alignment of *FoEGR6* from *F. oxysporum***.** GMQE: Global model quality.

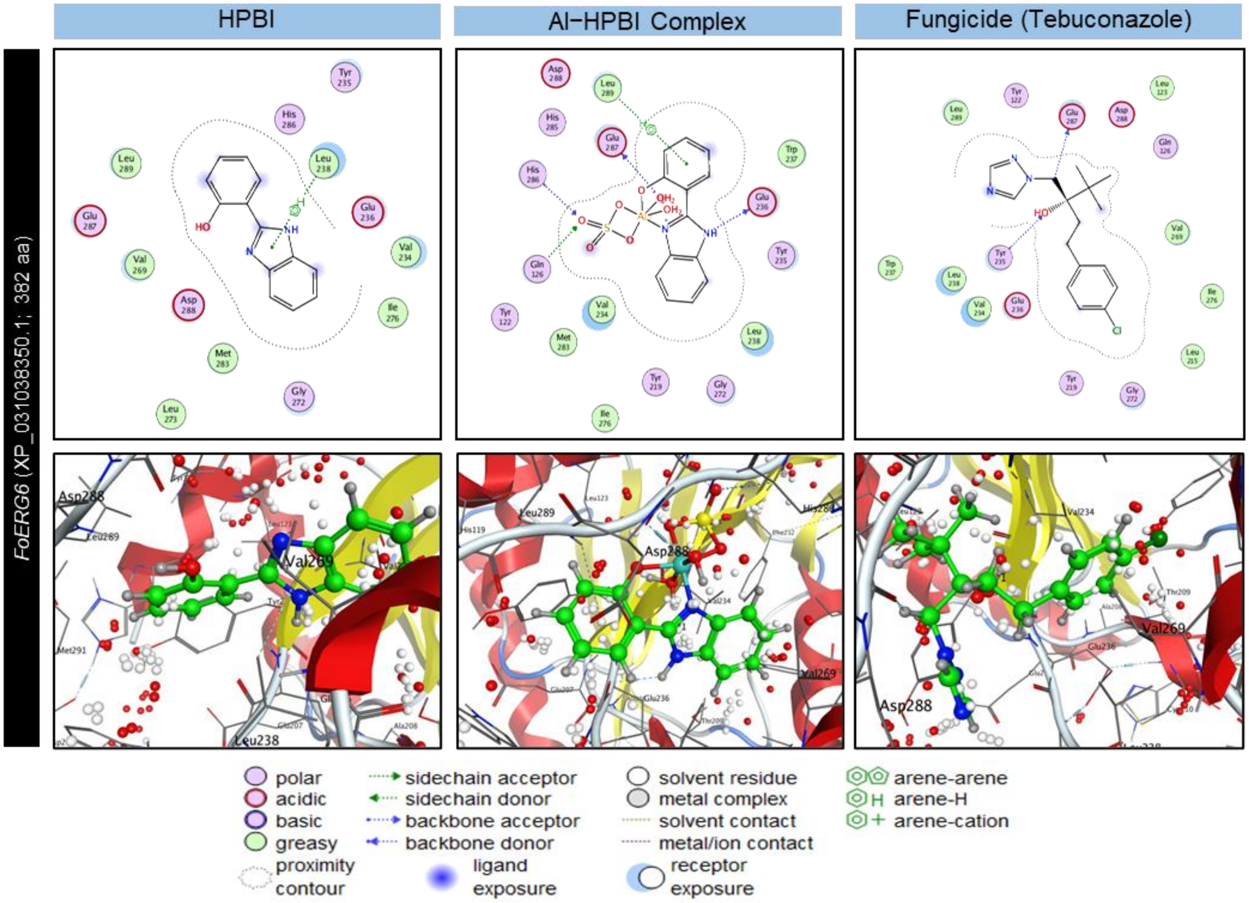

Supplementary Figure S3. Two-dimensional (2D) and three-dimensional (3D) docking interaction of HPBI, Al–HPBI Complex, and Fungicide (Tebuconazole) with 24-C-methyltransferase from *F. oxysporum* (*FoEGR6*; XP_031038350.1).
